# The effect and mechanism of Fushen Granule on gut microbiome in the prevention and treatment of chronic renal failure

**DOI:** 10.3389/fcimb.2023.1334213

**Published:** 2024-01-11

**Authors:** Lin Wang, Ao Xu, Jinxiang Wang, Guorong Fan, Ruiqi Liu, Lijuan Wei, Ming Pei

**Affiliations:** ^1^ Nephrology Department, First Teaching Hospital of Tianjin University of Traditional Chinese Medicine, Tianjin, China; ^2^ Graduate School, Tianjin University of Traditional Chinese Medicine, Tianjin, China; ^3^ Guangdong Provincial Key Laboratory of Digestive Cancer Research, Precision Medicine Center, Scientific Research Center, The Seventh Affiliated Hospital, Sun Yat-Sen University, Shenzhen, China; ^4^ Nephrology Department, Tianjin Academy of Traditional Chinese Medicine Affiliated Hospital, Tianjin, China

**Keywords:** chronic renal failure, intestinal microecology, fecal microbiota transplantation, renal-intestinal axis, Fushen Granule

## Abstract

**Background:**

Fushen Granule is an improved granule based on the classic formula Fushen Formula, which is used for the treatment of peritoneal dialysis-related intestinal dysfunction in patients with end-stage renal disease. However, the effect and mechanism of this granule on the prevention and treatment of chronic renal failure have not been fully elucidated.

**Methods:**

A 5/6 nephrectomy model of CRF was induced and Fushen Granule was administered at low and high doses to observe its effects on renal function, D-lactate, serum endotoxin, and intestinal-derived metabolic toxins. The 16SrRNA sequencing method was used to analyze the abundance and structure of the intestinal flora of CRF rats. A FMT assay was also used to evaluate the effects of transplantation of Fushen Granule fecal bacteria on renal-related functional parameters and metabolic toxins in CRF rats.

**Results:**

Gavage administration of Fushen Granule at low and high doses down-regulated creatinine, urea nitrogen, 24-h urine microalbumin, D-lactate, endotoxin, and the intestinal-derived toxins indophenol sulphateand p-cresol sulphate in CRF rats. Compared with the sham-operated group in the same period, CRF rats had a decreased abundance of the firmicutes phylum and an increased abundance of the bacteroidetes phylum at the phylum level, and a decreasing trend of the lactobacillus genus at the genus level. Fushen Granule intervention increased the abundance of the firmicutes phylum, decreased the abundance of the bacteroidetes phylum, and increased the abundance of the lactobacillus genus. The transplantation of Fushen Granule fecal bacteria significantly reduced creatinine(Cr), blood urea nitrogen(Bun), uric acid(UA), 24-h urinary microalbumin, D-lactate, serum endotoxin, and enterogenic metabolic toxins in CRF rats. Compared with the sham-operated group, the transplantation of Fushen Granule fecal bacteria modulated the Firmicutes and Bacteroidetes phyla and the Lactobacillus genus.

**Conclusion:**

Fushen Granule improved renal function and intestinal barrier function by regulating intestinal flora, inhibiting renal fibrosis, and delaying the progression of chronic renal failure.

## Graphical abstract

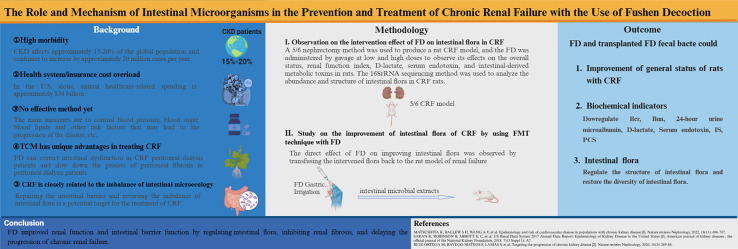

## 1 Introduction

Chronic renal failure (CRF) is a progressive disease resulting from various chronic kidney diseases (CKD), leading to abnormalities in kidney tissue structure and function until eventual failure (Fang et al., 2023). Its development into the final stage, end-stage renal disease (ESRD), poses a significant threat to human health and consumes considerable health resources(Wouters et al., 2015) (Dai et al., 2021; Wouk, 2021). Statistics show that CKD affects approximately 15-20% of the global population (Matsushita et al., 2022), with the number of cases increasing by about 20 million per year. The prevalence of CRF stands at 15.1% among adults in the US and 10.8% in China (Wang et al., 2021). As a late complication of CKD (Webster et al., 2017), ESRD is a major disease with high morbidity and mortality rates worldwide (Zhang et al., 2012; Liyanage et al., 2015). The cost of treating ESRD is currently staggering; in the US alone, it is estimated to be around US$34 billion per year (Saran et al., 2018). The burden of CRF on healthcare systems and health insurance costs has become a tremendous challenge for public health systems worldwide. Therefore, determining how to delay the disease process and improve patients’ quality of life during the chronic kidney failure phase is a crucial issue in preventing and treating chronic kidney disease and end-stage renal disease.

The treatment of CRF still has no specific method in the international medical community, mainly to control protein intake, anti-oxidative stress, blood pressure, blood glucose and lipid control and other risk factors that may lead to the progression of the disease to slow down the deterioration (Ruiz-Ortega et al., 2020; Faria and de Pinho, 2021); Although there has been great progress in the study of molecular mechanisms and related markers, the clinical translation is poor. In recent years, more and more studies have demonstrated that the development of CRF is closely related to the imbalance of intestinal microecology, and that disorders of intestinal flora and disruption of intestinal barrier structure and function play an important role in disease progression (Yang et al., 2018; Pluznick, 2020). Intestinal dysfunction can be seen in the early stages of CRF, mainly in the form of digestive symptoms such as impaired digestion and absorption in the intestine, as well as varying degrees of impairment of intestinal barrier function. The presence of the intestinal barrier prevents bacteria, pathogens and other harmful substances in the intestinal lumen from entering the bloodstream to maintain the stability of the internal environment. After the intestinal barrier is damaged, endotoxins and intestinal flora can enter the blood circulation, resulting in enterogenic endotoxemia, which aggravates the micro-inflammatory state of the body. CRF and peritoneal dialysis patients are combined with varying degrees of endotoxemia (Gonçalves et al., 2006; Szeto et al., 2008; Feroze et al., 2012), and uremic rats are more prone to lateral translocation of intestinal bacteria (de Almeida Duarte et al., 2004), this is a reflection of the impaired intestinal barrier in CRF. In addition, the accumulation of intestinal proteins in CRF alters the structure of the intestinal flora and increases the number of enteric proteolytic bacteria. Tryptophan is fermented in the intestine to produce indole, which is absorbed into the blood via the intestine and converted to IS in the liver; tyrosine and phenylalanine are fermented in the intestine to produce p-cresol, which is converted to PCS in the intestinal epithelium (Liabeuf et al., 2011). The latter is converted to PCS in the intestinal epithelium. Since IS and PCS are bound to albumin in the blood and cannot be excreted through dialysis, their accumulation in the body can accelerate the progression of renal failure by aggravating glomerulosclerosis and interstitial fibrosis (Miyazaki et al., 1997; Gelasco and Raymond, 2006; Watanabe et al., 2013). Therefore, repairing the intestinal barrier and reversing the imbalance of intestinal flora are considered as potential therapeutic targets and new targets for intervention in the treatment of CRF.

Traditional Chinese medicine (TCM) holds a unique advantage in the treatment of CRF. CRF involves a complex interplay among various organs, with a particularly intricate relationship between the spleen and kidney systems in TCM. The fundamental mechanism underlying CRF is often described as “deficiency of the spleen and kidney, accumulation of turbid toxins.” Guided by this medical principle, the Department of Nephrology at the First Affiliated Hospital of Tianjin University of Traditional Chinese Medicine has tailored its approach to the pathogenic patterns of CRF. Draw upon extensive clinical practice and years of specialized case data, and referencing the optimized compilation and efficacy validation of nationwide TCM protocols for CRF, this has ultimately evolved into the “Fushen Formula.” This formula serves as the basis for a corresponding composite herbal preparation named “Fushen Granule.” The granule preparation has received hospital formulation approval (Batch Number: 140928) and is primarily employed to treat intestinal dysfunctions related to peritoneal dialysis in ESRD patients. It has been shown to achieve definite therapeutic effects in clinical practice. In clinical application, the treatment group receiving Fushen Granule demonstrated superior improvement in renal function and reduction in blood creatinine(BCR) and blood urea nitrogen(BUN) levels compared to the control group, with no adverse reactions observed (Sun and Liu, 2010). Among elderly CKD stage 4 patients, oral administration of Fushen Granule significantly enhanced kidney function and nutritional status, ameliorated symptoms, and elevated quality of life (Yang and Yang, 2015). Another study revealed that the combination of blood purification and oral administration of Fushen Granule for treating CRF significantly improved kidney function and quality of life, reduced hospitalization time, lowered mortality rates, and alleviated economic burdens (Lei, 2016). Furthermore, the comprehensive efficacy of oral administration of Fushen Granule combined with acupuncture at specific points was found to surpass that of conventional acupuncture alone in CKD stage 4 patients (Zhang and Yang, 2016). Building upon clinical practice, fundamental research on Fushen Granule has also been initiated. Results from basic experiments demonstrate that Fushen Granule can slow down the progression of renal function impairment in a rat model of CRF. It reduces the expression of mild inflammatory factors (such as hs-CRP, IL-6, and TNF-α), lowers blood urea nitrogen(BUN) and blood creatinine(BCR) levels, thereby improving kidney function and delaying the progression of renal deterioration (Zhang and Yang, 2018a; Zhang and Yang, 2018b). Fushen Granules are not only suitable for patients with CRF, but also for conditions such as peritoneal dialysis in CRF and renal interstitial fibrosis (Zhao et al., 2022). A multicenter clinical study involving 240 peritoneal dialysis patients over a 6-month period demonstrated significant enhancements attributed to Fushen Granules. These enhancements include improved TCM syndromes, enhanced dialysis efficiency, increased removal of toxins and excess water, slowed decline of residual kidney function, and an overall improvement in quality of life (Yang et al., 2012a; Yang et al., 2013). Additionally, Fushen Granules exhibited positive effects on the prognosis of peritonitis (Jiang et al., 2020a), gastrointestinal function in peritoneal dialysis patients (Chen, 2016), and maintenance of protein nutritional status (Yang et al., 2014; Lei and Yang, 2018; Lei et al., 2018a). In peritoneal dialysis rats, research reveals that Fushen Granules improve renal function damage through multiple pathways, including modulating Glo-1 expression levels and reducing serum AGEs accumulation, thereby safeguarding residual kidney function(Tang et al., 2017; Tang et al., 2018b). Furthermore, it alleviates ultrafiltration volume and glucose transport, enhancing peritoneal dialysis efficacy(Yang et al., 2021b). Fundamental research also indicates that Fushen Granules regulate Dcn, inhibit activation of the TGF-β pathway, disrupt extracellular matrix accumulation, modulate glucose and lipid metabolism, suppress expression of fibrosis-promoting factors (such as TGF-β1, TGF-βRII, CTGF, IL-6, CTGF, VEGF), and elevate expression of anti-fibrotic factors (HGF, BMP-7), thereby restraining peritoneal collagen formation and inhibiting mesothelial cell transdifferentiation. As a result, it slows the progression of peritoneal fibrosis (Yang et al., 2012b; Dou, 2015; Lei et al., 2018b; Lei et al., 2018c; Jiang et al., 2020b; Li et al., 2020; Yang et al., 2021a). Additionally, Fushen Granules reduce the accumulation of pro-oxidative factors like MG and MDA, upregulate the expression of the antioxidant GSH, subsequently ameliorating anemic conditions in CRF peritoneal dialysis rats (Tang et al., 2018a).

Fecal microbiota transplantation (FMT) has recently gained renewed clinical interest as a long-established therapy for rebuilding the intestinal flora. FMT, i.e. the isolation of functional donor or autologous intestinal flora and transplantation into the patient’s gut, has been applied to treat a range of gastrointestinal/non-gastrointestinal disorders associated with intestinal flora and is a breakthrough medical advance in recent years (Cui et al., 2016). In addition, FMT is also used in the renal field for kidney transplantation (Ahmad and Bromberg, 2016). Although the correlation between intestinal flora and CKD/CRF is currently a hot topic of research, the use of FMT in the treatment of CKD/CRF has not yet been reported. Therefore, within the scope of affirming Fushen Granules’ ability to retard peritoneal fibrosis progression, enhance dialysis efficacy, and improve quality of life for peritoneal dialysis patients—particularly in ameliorating intestinal functional disorders—we have integrated Fushen Granules with FMT. This integration enables a more profound and direct impact of post-intervention microbiota reinfusion on intestinal microbiota dynamics. From this perspective, the pivotal role and significance of gut microbiota in CRF are harnessed, establishing novel avenues and intervention targets for preventing and treating CRF progression.

## 2 Materials and methods

### 2.1 Preparation of the experimental drug, Fushen Granule

Fushen Granules (Batch number: 140928) were provided by the First Teaching Hospital of Tianjin University of Traditional Chinese Medicine (Tianjin, China) in strict compliance with the Good Manufacturing Practice and Good Laboratory Practice guidelines for Pharmaceutical Manufacturers. Fushen Granules are primarily composed of eight different herbal ingredients. The names of these herbs have been revision in the plant list (http://www.theplantlist.org), and the details can be found in **Table 1**. Through UPLC/Q-TOF analysis, the main active components of the Fushen Granules were characterized. We have preliminarily identified 55 different compounds in FSG, as detailed in the attached document. The granules were dissolved in distilled water, and the dosage for rats was adjusted based on the body surface area of humans, with equal amounts administered as low doses and 10 times the amount given as high doses.

**Table 1:** The constituents of Fushen Granule.

Num	Scientific name	Material	Latin name	Chinese name	Mass Ratio
1	Astragalus membranaceus	Root	*Hedysarum Multijugum* Maxim	Huangqi	15g
2	Angelica sinensis	Root	*Angelica sinensis* (Oliv.) Diels	Danggui	10g
3	Epimedium brevicornu Maxim	Root	*Epimedium brevicornu* Maxim	Xianlingpi	15g
4	Citrus reticulata Blanco	outer pericarp	*Citrus reticulata* Blanco	Chenpi	10g
5	Pinellia ternata	Roots and rootstalk	*Pinellia ternata* (Thunb.) Makino	Banxia	15g
6	Salvia miltiorrhiza Bunge	Roots and rootstalk	*Salvia miltiorrhiza* Bunge	Danshen	30g
7	Rheum	Roots and rootstalk	*Rheum palmatum* L.	Dahuang	10g
8	Siebold	Winged shoots or branch wings	*Euonymus alatus* (Thunb.) Siebold	Guijianyu	30g

### 2.2 Laboratory animals

Specific pathogen-free (SPF) healthy male Sprague-Dawley (SD) rats (weight 180 ± 20g) were purchased from the Experimental Animal Centre of the Chinese People’s Liberation Army Academy of Military Medical Sciences. They were housed in the Experimental Animal Centre of Tianjin University of Chinese Medicine. Throughout the experiment, the rats were fed normal chow (no yeast, no probiotics) and had ad libitum access to water. All animal procedures were conducted in accordance with the NIH Guide for the Care and Use of Laboratory Animals and the Chinese Laboratory Animal Management Methods. The ethical approval for this study was No. IRM-DWLL-2020100.

### 2.3 Experimental reagents and apparatus

The following reagents and apparatus were used in the study: Rat Indoxyl Sulfate (IS) ELISA Kit (Jianglai Bio, JL44174), Rat P-Cresol Sulfate (PCS) ELISA Kit (Jianglai Bio, JL48836), Lactate Test Kit (Nanjing Jiancheng, A019-2-1), Endotoxin Assay Kit (Xiamen Horseshoe Crab Bio, EC80545), Urea Nitrogen Assay Kit (Changchun Huili, C010), Creatinine Assay Kit (Changchun Huili, C074), Uric Acid Assay Kit (Changchun Huili, C075), and DNA Isolation Kit (MoBio, Carlsbad, CA, USA). An Illumina HiSeq platform was provided by Novogene Bioinformatics Co. Ltd., Beijing, China.

Equipment used in the study included a vertical refrigerated display cabinet (Star), a horizontal freezer (Meiling), an electronic balance (Mettler-Toledo Instruments (Shanghai) Co., Ltd.), a high-speed tissue grinder (Servicebio), a benchtop high-speed frozen centrifuge (ThermoFisher), and an enzyme-linked immunosorbent assay (BioTek).

## 3 Methods

### 3.1 Chronic renal failure rat model construction and sample collection

In this study, a 5/6 nephrectomy was performed to establish a chronic renal failure(CRF) model in rats. The left kidney was initially resected by 5/6 nephrectomy, and after a 1-week recovery period, the right kidney was completely resected. Blood and urine biochemical tests were conducted at the end of the modeling period. Fresh feces from each group were collected in sterile lyophilized tubes, immediately placed in liquid nitrogen, and then stored at -80°C.

### 3.2 Observation on the intervention effect of Fushen Granule on intestinal flora in CRF

For *in vivo* studies, 150 specific pathogen-free (SPF) healthy male Sprague-Dawley (SD) rats were acclimatized for 1 week. The rats were randomly divided into the following groups: 30 rats in the sham-operated group (Group A), 30 rats in the sham-operated Chinese medicine group (Group B), and 90 rats in the modeling group. The modeling group was further divided into a low-dose Chinese medicine group (Group C), a high-dose Chinese medicine group (Group D), and a control model group (Group E), with 30 animals in each group. Groups B and C received a low dose of Fushen Granule via gavage; Group D received 10 times the high dose of Fushen Granule via gavage; Groups A and E were given 2ml·d-1 distilled water via gavage. Six animals from each group were euthanized for examination at the beginning of drug intervention, at the end of the second week, and at the end of the fourth week. All remaining animals were euthanized at the end of the sixth week.

### 3.3 Fecal microbiota transplant experiment

After 1 week of acclimatization, 55 specific pathogen-free (SPF) healthy male Sprague-Dawley (SD) rats were randomly divided into a 5/6 nephrectomized kidney failure rat model recipient group (15 x 3) and a donor rat group (5 x 2, approximately 1/3 the number of the recipient group). The donor group was further divided into the Fushen Granule donor group and the normal donor group, while the recipient group was divided into the model group + Fushen Granule donor FMT group, the model group + normal donor FMT group, and the model group + saline sham operation group. In the Fushen Granule donor group, a low dose of Fushen Granule was administered for 6 weeks from the first day of right nephrectomy, based on the effect of the previous experimental intervention. No intervention was made in the normal donor group. Fresh, uncontaminated feces were collected daily, and intestinal microbial extracts were prepared in saline. Fecal microbiota transplantation(FMT) was completed within 4 hours of feces collection. The recipient groups, model group + Fushen Granule donor and model group + normal donor, underwent FMT with intestinal microbial extracts from the Fushen Granule donor group and the normal donor group, after determining successful modeling through dynamic observation of kidney function. The model group + saline sham group underwent a procedure with an equal amount of saline equivalent as the control group.

### 3.4 Observation indicators

The animals were dynamically observed, and their body weight was assessed every 5 days. The focus was on the general status of the rats at the beginning of drug intervention and at the end of the 2nd, 4th, and 6th weeks (general status in terms of behavioral activity, body weight, phenotypic signs, and survival rate), renal function indicators (Cr, BUN, UA, eGFR, and urinary microalbumin), serum endotoxin, D-lactate, metabolic toxins of intestinal origin (IS, PCS), and analysis of intestinal bacterial diversity.

### 3.5 Analysis of intestinal bacterial diversity

In this study, 16S rRNA was used to assess intestinal bacterial diversity, but the timing of fecal sample collection differed between the two experiments. Fresh fecal samples were collected at around 10 am at the beginning of the intervention and at the end of the second, fourth, and sixth weeks. Fecal samples were collected from the three model groups at two time points: before the start of the FMT experiment and 15 days after the transplantation (Note: The feces were collected for the same amount of time each instance). Fresh fecal samples collected were immediately frozen in liquid nitrogen for 5 minutes and then stored at -80°C. DNA was extracted from feces using the Power Fecal DNA Isolation Kit (MoBio, Carlsbad, CA, USA). DNA was recovered using 30 mL of the buffer included in the kit. Sequencing principles were used for synthetic sequencing with the Illumina HiSeq platform (Novogene Bioinformatics Co., Beijing, China). Taxonomic composition of the flora was analyzed to evaluate bacterial abundance and compositional diversity, using phylum, order, family, and genus classifications.

### 3.6 Statistical analysis

SPSS 19.0 for Windows statistical software was used for data analysis. Data were expressed as mean ± SEM relative to the number of samples in each group (n). Analysis of variance (ANOVA), Wilcoxon rank-sum test, Tukey’s t-test, and Student’s t-test were used to determine statistical significance between multiple treatment groups. The Kaplan-Meier survival test was used to analyze survival rates. Results with p< 0.05 were considered statistically significant.

## 4 Results

### 4.1 Effect of Fushen Granule on the status of rats with CRF

#### 4.1.1 General state

During the modeling phase, rats in the model group typically exhibited irritability, emotional instability, and agitated reactions when injected or given the drug via gavage. Over time, rats in the sham-operated group gained weight, appeared in good spirits, moved freely, were responsive, and had well-groomed, moisturized fur. In contrast, rats in the model group gradually lost weight, ate less than the sham-operated group, appeared more depressed, squinted, were less active, less responsive, and had disheveled and unkempt fur. Rats in the drug intervention group demonstrated better performance in terms of mental status, body weight, activity, responsiveness, and fur appearance after treatment, but still showed slight differences compared to the sham-operated group.

#### 4.1.2 Observation of renal function indicators

Compared with the sham-operated group, the model group showed a statistically significant increase in Cr, BUN, and 24-h urinary microalbumin at weeks 2, 4, and 6 (P< 0.01), indicating successful modeling of renal failure (see **Table 2**). UA also tended to increase, but the difference was not statistically significant. Compared with the model group, the 24-h urine microalbumin in the Chinese medicine low-dose group decreased significantly at weeks 2 and 4 (P< 0.001), BUN decreased significantly at week 4 (P< 0.05), and Cr decreased significantly at week 6 (P< 0.05). The Chinese medicine high-dose group showed significant decreases in Cr and 24-h urine microalbumin at weeks 2, 4, and 6 (P< 0.01), and BUN at week 4 (P< 0.05) (**Table 2**). In conclusion, the herbal intervention group had a positive effect on the renal function indices of rats with CRF, and the efficacy of the high-dose herbal group was better.

**Table 2:** Indicators of kidney function.

Group	Point in time	Cr (mmol/L)	BUN (µmol/l)	UA (μmol/l)	24-h urine microalbumin (mg/L)
Sham-operated Group A	Initial	48.25 ± 4.28	18.29 ± 2.15	108.35 ± 4.79	23.28 ± 4.82
	2 weeks	42.45 ± 3.35	14.28 ± 2.23	104.22 ± 16.38	26.75 ± 3.24
	4 weeks	65.59 ± 10.07	16.58 ± 0.61	107.53 ± 20.37	29.36 ± 4.91
	6 weeks	74.31 ± 5.05	38.66 ± 1.07	117.16 ± 12.77	25.18 ± 3.52
Fushen Granule low dose control (Group B)	Initial	50.23 ± 5.97	20.34 ± 2.46	110.25 ± 5.32	25.17 ± 3.34
	2 weeks	55.9 ± 5.23	13.98 ± 2.80	121.00 ± 20.57	25.37 ± 5.82
	4 weeks	65.72 ± 9.82**	16.04 ± 1.16	109.29 ± 14.96	27.54 ± 3.26
	6 weeks	72.34 ± 8.14	38.47 ± 0.67	139.85 ± 40.34	24.34 ± 2.84
Model (Group E)	Initial	47.93 ± 4.74	19.23 ± 2.73	115.32 ± 5.28	27.43 ± 3.44
	2 weeks	109.23 ± 4.56***	50.24 ± 4.56***	110.25 ± 11.39	128.37 ± 12.37***
	4 weeks	103.75 ± 2.57**	45.47 ± 0.95**	104.42 ± 16.04	379.85 ± 28.37***
	6 weeks	107.79 ± 12.37**	44.33 ± 2.1**	127.40 ± 18.15	583.92 ± 45.29***
Fushen Granule at low doses (Group C)	Initial	51.2 ± 5.22	20.12 ± 1.94	109.73 ± 4.91	24.82 ± 4.19
	2 weeks	106.78 ± 1.25	45.91 ± 3.12	108.13 ± 26.28	88.24 ± 6.74###
	4 weeks	96.46 ± 3.67**	25.48 ± 2.63#	107.68 ± 24.15	128.49 ± 18.58###
	6 weeks	95.41 ± 3.91**#	44.63 ± 1.45**	136 ± 34.74	73.95 ± 10.28###
Fushen Granule at high doses (Group D)	Initial	49.37 ± 4.60	18.32 ± 2.31	111.20 ± 4.83	26.37 ± 2.58
	2 weeks	95.24 ± 3.13##	44.15 ± 4.50	103.67 ± 30.12	75.63 ± 6.53###
	4 weeks	85.1 ± 9.7**##	41.69 ± 0.9**#	120.97 ± 24.09	102.53 ± 13.45###
	6 weeks	90.41 ± 9.77**##	44.03 ± 2.24**	119.87 ± 15.49	58.24 ± 8.94###

Compared to the sham-operated group: * indicates p< 0.05, ** indicates p< 0.01, *** indicates p< 0.001; compared to the model group, # indicates p< 0.05, ## indicates p< 0.01, ### p< 0.001.

#### 4.1.3 Observation of serum endotoxins and metabolic toxin-like indicators of enteric origin

Compared with the sham-operated group, rats in the model group showed significantly higher IS, PCS, D-lactic acid, and endotoxin levels at weeks 2, 4, and 6 (P< 0.05), indicating successful modeling of renal failure. Compared with the model group, the Chinese medicine low-dose group showed significant decreases in IS, PCS, D-lactate, and endotoxin at weeks 2, 4, and 6 (P< 0.05). The high-dose group of Chinese medicine showed a significant decrease in PCS (P< 0.01) at the beginning of the drug intervention and in IS, PCS, D-lactic acid, and endotoxin at weeks 2, 4, and 6 (P< 0.05) (**Table 3**). This indicates that both the low and high dose groups of Chinese medicine demonstrated improvement in D-lactate, endotoxin, and enteric-derived metabolic toxin-like indicators. Compared with the low-dose group, the high-dose group generally had a better effect on D-lactate, endotoxin, and intestinal-derived metabolic toxins.

**Table 3:** Indicators for D-lactate, endotoxin and enteric-derived metabolic toxin categories.

Group	Point in time	Metabolic toxins of enteric origin		Serum endotoxin	
		IS (μg/ml)	PCS (μg/ml)	D-Lactic acid (μg/ml)	Endotoxin (EU/ml)
Sham-operated (Group A)	Initial	1.02 ± 0.12	0.03 ± 0.01	7.68 ± 0.27	0.019 ± 0.003
	2 weeks	1.35 ± 0.34	0.05 ± 0.01	7.59 ± 0.31	0.029 ± 0.004
	4 weeks	1.66 ± 1.12	0.08 ± 0.01	7.51 ± 0.21	0.027 ± 0.002
	6 weeks	1.71 ± 0.47	0.13 ± 0.07	7.66 ± 0.30	0.032 ± 0.004
Fushen Granule low dose control (Group B)	Initial	0.85 ± 0.23	0.02 ± 0.01	7.52 ± 0.31	0.021 ± 0.002
	2 weeks	0.90 ± 0.11	0.04 ± 0.01	7.45 ± 0.36	0.025 ± 0.003
	4 weeks	0.96 ± 0.47	0.07 ± 0.01	7.22 ± 0.25	0.026 ± 0.006
	6 weeks	3.04 ± 0.6	0.06 ± 0.02	7.82 ± 0.48	0.021 ± 0.001
Model (Group E)	Initial	1.05 ± 0.19	0.05 ± 0.01*	7.61 ± 0.27	0.023 ± 0.003
	2 weeks	3.67 ± 0.65***	0.43 ± 0.11***	15.92 ± 0.28***	0.316 ± 0.027***
	4 weeks	6.02 ± 1.58**	0.58 ± 0.38**	20.31 ± 0.48***	0.429 ± 0.031***
	6 weeks	6.35 ± 2.24**	1.74 ± 0.34*	14.76 ± 0.34***	0.496 ± 0.038***
Fushen Granule at low doses (Group C)	Initial	0.98 ± 0.10	0.04 ± 0.01	7.76 ± 0.24	0.026 ± 0.003
	2 weeks	2.59 ± 0.35##	0.20 ± 0.04###	10.22 ± 0.47###	0.209 ± 0.023###
	4 weeks	3.89 ± 0.78**##	0.26 ± 0.1	9.03 ± 1.02###	0.297 ± 0.027###
	6 weeks	3.54 ± 1.97#	0.3 ± 0.23#	11.76 ± 0.13###	0.234 ± 0.025###
Fushen Granule at high doses (Group D)	Initial	0.95 ± 0.15	0.02 ± 0.01##	7.53 ± 0.25	0.025 ± 0.001
	2 weeks	2.48 ± 0.32##	0.13 ± 0.02###	9.84 ± 0.52###	0.174 ± 0.018###
	4 weeks	3.55 ± 0.79*##	0.15 ± 0.04#	10.79 ± 0.63###	0.216 ± 0.023###
	6 weeks	3.19 ± 1.56#	0.39 ± 0.24#	12.01 ± 0.44###	0.183 ± 0.017###

Compared to the sham-operated group: * indicates p< 0.05, ** indicates p< 0.01, *** indicates p< 0.001; compared to the model group, # indicates p< 0.05, ## indicates p< 0.01, ### p< 0.001.

#### 4.1.4 Analysis of intestinal bacterial diversity

##### 4.1.4.1 Sequencing data quality and OTU analysis

**Figure 1:**
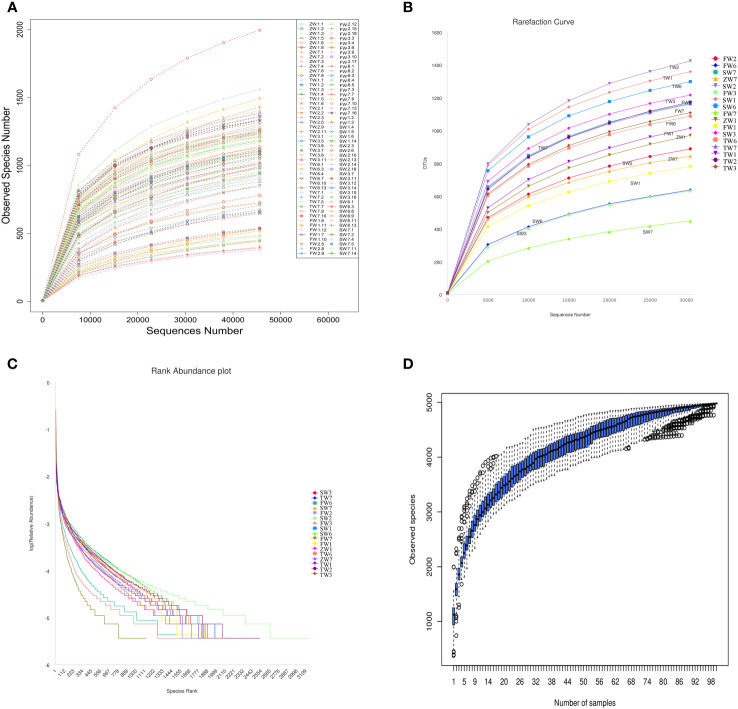
Analysis of intestinal bacterial diversity. **(A, B)** Sparsity curve of the sample to be tested; **(C)** Rank Abundance curve; **(D)** Cumulative box plot of species.

The IonS5TMXL sequencing platform was utilized to analyze the diversity of intestinal bacteria. Using a single-end sequencing method, an average of 83,925 reads per sample were measured by shear filtering of Reads, and an average of 78,965 valid data were obtained after quality control, with a quality control efficiency of 94.15%. The sequences were clustered into OTUs (Operational Taxonomic Units) with 97% agreement, yielding a total of 4,955 OTUs. The OTUs sequences were then analyzed against the Silva132 database for species annotation to understand the composition and differences between intestinal flora. A total of 4,955 OTUs were counted according to different taxonomic levels, of which the number of OTUs that could be annotated to the database was 4,954 (99.98%). Dilution curves were constructed by the number of OTUs detected at each sequencing depth of the experimental data, and it was found that as the sequencing depth gradually increased, the sparsity curve also gradually smoothed out, indicating that the current sequencing depth is essentially sufficient to reflect the microbial diversity contained in this community sample (**Figures 1A, B**). The smoothness of the Rank Abundance curve reflects the uniformity of the species distribution, and from the figure (**Figure 1C**), it can be observed that the distribution of microbial species contained in this sequenced sample was poorly distributed. Species accumulation box plots were used for sample size and diversity analysis of the biota. As seen in the box line plot (**Figure 1D**), the box positions no longer rose sharply but leveled off as the sample size expanded, indicating that the current sample size was abundant and adequate, and largely representative of the community’s flora characteristics.

**Figure 2:**
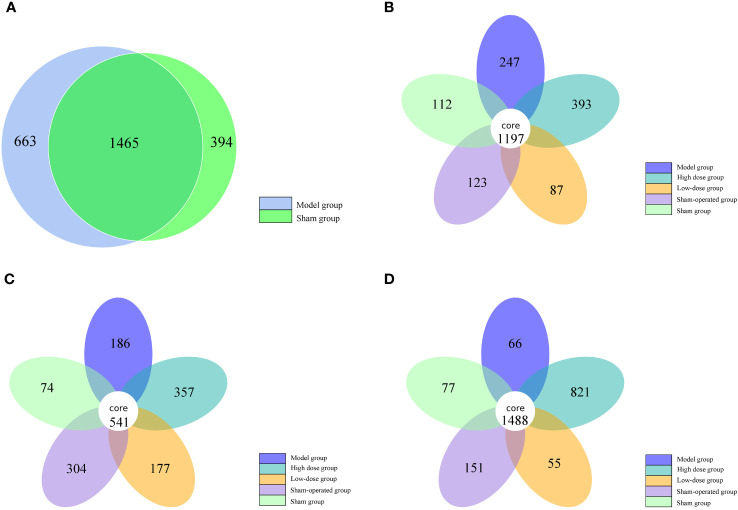
Number of OTUs of intestinal flora in five groups of rats at 0, 2, 4 and 6 weeks. **(A)** 0 weeks, **(B)** 2 weeks, **(C)** 4 weeks, **(D)** 6 weeks.

By clustering, sequences can be grouped into various categories based on their similarity to each other, with each category representing a taxonomic OTU. OTUs with a 97% similarity level are typically analyzed for bioinformatic statistics. In this study, we analyzed intestinal bacterial diversity in 5/6 nephrectomized rats. The results showed that the number of OTUs shared between the model and sham-operated groups was highest at week 0 (1465). However, the number of OTUs shared by the five groups gradually decreased at weeks 2 and 4, with 1197 and 541, respectively. This indicates that 5/6 nephrectomy can cause a decrease in the number of OTUs in the group. In terms of unique OTUs, the model group had 247, 186, and 66 unique OTUs at weeks 2, 4, and 6, respectively. The Chinese medicine high-dose treatment group had 393, 357, and 821 unique OTUs, while the low-dose treatment group had 87, 177, and 55 unique OTUs in that order. These findings suggest that Fushen Granule may alleviate intestinal flora damage and increase the number of OTUs in rats with 5/6 nephrectomy to some extent. Compared with the same time points, the herbal high-dose treatment groups at weeks 2, 4, and 6 contained the highest number of unique OTUs. This indicates that the Chinese medicine high-dose group was more effective in restoring intestinal flora diversity in 5/6 nephrectomized rats than the low-dose treatment group (**Figure 2**).

##### 4.1.4.2 Species annotation and taxonomic analysis

**Figure 3:**
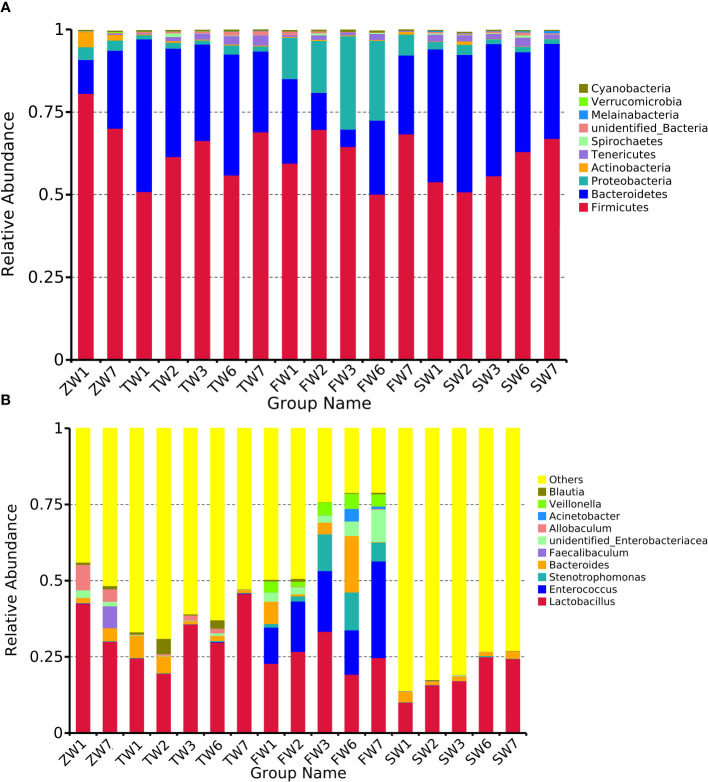
Top 10 relative abundance of intestinal flora in each group of rats **(A)** phylum level, **(B)** genus level. ZW indicates zero week, TW indicates two-week, FW indicates fourth week, SW indicates sixth week. 1 indicates model, 2 indicates fushen granule at high doses, 3 indicates fushen granule at low doses, 6 indicates sham-operated fushen granule control, 7 indicates sham-operated.

Species abundance tables at different taxonomic levels were generated using QIIME software, and then mapped into community structure at each taxonomic level of the samples using R language tools. The results showed that the dominant species at the phylum level were Firmicutes, Bacteroidetes, and Proteobacteria; at the genus level, the dominant species were Lactobacillus, Enterococcus, and Stenotrophomonas. The phylum Firmicutes and the phylum Bacteroidetes are the most abundant bacterial phyla in the intestinal flora, accounting for more than 90% of all bacteria. An analysis of the relative abundance of the intestinal flora at the phylum and genus taxonomic levels is shown in **Figure 3A, B**. In the sham-operated group, the levels of the two bacteria, Phylum Firmicutes and Phylum Bacteroidetes, were relatively stable and did not change over time. The relative abundance of Phylum Firmicutes was 70%, 69%, 68%, and 63%, and the relative abundance of Phylum Bacteroidetes bacteria was 24%, 24%, 22%, and 30% at the four time points of 0, 2, 4, and 6 weeks, respectively. The ratio of Phylum Firmicutes to Phylum Bacteroidetes bacteria remained roughly between 2 and 3. In contrast, in the 5/6 nephrectomy model group, the relative abundance of the Firmicutes phylum decreased, and the relative abundance of the Bacteroidetes increased, resulting in a decrease in the Firmicutes phylum/Bacteroidetes phylum ratio. Compared to the sham-operated group during the same period, the relative abundance of Firmicutes phylum bacteria in the model group decreased from 81% at week 0 to 54% at week 6, while the relative abundance of Bacteroidetes phylum increased from 10% at week 0 to nearly 40% at week 6, with a decreasing trend in the ratio of the two contents (**Table 4**). Compared with the model group, the Chinese medicine low-dose and Chinese medicine high-dose groups could differently reduce this alteration to some extent and restore the Firmicutes phylum and Bacteroidetes phylum composition ratio. For example, at the second week, the Firmicutes phylum and Bacteroidetes phylum ratio was 2.88 in the sham-operated group, and the ratio was 1.1, 2.27, and 1.88 in the model, low-dose, and high-dose Chinese medicine groups, respectively (**Table 5**). In terms of genus level, Lactobacillus was the main dominant genus, and compared with the sham-operated group during the same period, the level of Lactobacillus in the model group showed a decreasing trend, at 25% at 2 weeks, 23% at 4 weeks, and 10% at 6 weeks, respectively. Compared with the model group, the low dose of Chinese medicine and high dose of Chinese medicine treatment could increase the content of Lactobacillus to different degrees (**Table 6**).

**Table 4:** Levels of Firmicutes and Bacteroides of rats in sham-operated and model groups at 0, 2, 4 and 6 weeks.

Group	Time Point (weeks)	Firmicutes (%)	Bacteroides (%)	Firmicutes/Bacteroidetes ratio
Sham-operated	0	70	24	2.97
	2	69	24	2.88
	4	68	22	3.1
	6	63	30	2.1
Model	0	81	10	8.1
	2	51	46	1.11
	4	60	26	2.31
	6	54	40	1.35

**Table 5:** Levels of Firmicutes and Bacteroides of rats in each group at week 2.

Group	Firmicutes (%)	Bacteroidetes (%)	Firmicutes/Bacteroidetes ratio
Sham	69%	24%	2.88
Sham-operated	56%	37%	1.53
Model	51%	46%	1.11
Fushen Granule at low doses	66%	29%	2.27
Fushen Granule at high doses	62%	33%	1.88

**Table 6:** Levels of Lactobacillus of rats in each group at 2, 4 and 6 weeks.

Group	Lactobacillus 2 weeks	Lactobacillus 4 weeks	Lactobacillus 6 weeks
Sham	0.46	0.25	0.25
Sham-operated	0.30	0.19	0.25
Model	0.25	0.23	0.10
Fushen Granule at low doses	0.36	0.33	0.17
Fushen Granule at high doses	0.20	0.27	0.16

### 4.2 Using FMT as a technical platform to study the Improvement of Intestinal Flora in CRF with Fushen Granule

#### 4.2.1 General state observation

The rats in the control donor group had significantly increased body weight, good mental condition, free movement, responsiveness, and neat and moist hair. In contrast, the rats in the model group gradually lost weight, ate less, appeared more depressed, squinted, were less active, less responsive, and had loose body hair and shaggy fur. The Fushen Granule donor group or FMT group had a better mental status, weight, activity, reaction, and coat color after treatment, but was still worse than the control group.

#### 4.2.2 Observation of renal function indicators

Compared with the normal donor group, Cr, BUN, UA, and 24-h urine microalbumin were significantly higher in the nephrectomy + saline group at weeks 2, 4, and 6, with statistically significant differences (P< 0.05), indicating successful modeling of renal failure. Compared with the nephrectomy + saline group, BUN and 24-h urine microalbumin decreased significantly (P< 0.05) at week 2, and Cr, BUN, UA, and 24-h urine microalbumin also decreased significantly (P< 0.05) at weeks 4 and 6 in the nephrectomy + Fushen Granule group. In contrast, BUN, UA, and 24-h urine microalbumin also decreased significantly in the nephrectomy + normal f group at week 2 (P< 0.05), and Cr, BUN, UA, and 24-h urine microalbumin decreased significantly at weeks 4 and 6 (P< 0.05). The improvement in 24-h urinary microalbumin at weeks 2, 4, and 6 did not significantly differ between the kidney cut + Fushen Granule group and the kidney cut + normal f group (P > 0.05) (**Table 7**).

**Table 7:** Indicators of renal function.

Group	Point in time	Cr	Bun	UA (μmol/L)	24-hour urine microalbumin (mg/L)
Normal Donor	Initial	44.39±0.81	20.43±0.44	102.27±1.08	20.28±2.89
	2 weeks	47.80±3.55	23.53±2.35	112.23±9.48	23.57±2.74
	4 weeks	45.89±0.98	22.89±0.95	104.83±2.04	28.32±2.53
	6 weeks	45.89±0.98	25.93±4.39	128.32±8.85	30.28±2.73
Kidney cut + saline	Initial	42.34±1.97	24.45±0.73	105.26±1.15	22.53±4.20
	2 weeks	95.78±1.96^**^	37.20±0.28^*^	145.60±1.19^**^	288.34±21.49^***^
	4 weeks	110.39±26.23^**^	33.33±8.98^*^	146.46±2.00^**^	482.95±32.94^***^
	6 weeks	110.39±26.23^**^	55.82±2.89^*^	190.33±45.55^**^	638.29±44.76^***^
Kidney cut + Fushen Decoction donor	Initial	45.12±2.49	20.81±0.64	105.87±2.73	21.15±2.46
	2 weeks	55.78±3.61^###^	26.06±3.51^#^	121.37±6.30^###^	75.24±7.93^###^
	4 weeks	38.15±1.22^###^	32.58±2.09	130.70±20.98^###^	129.27±10.89^###^
	6 weeks	38.15±1.22^###^	29.70±3.99^#^	128.50±5.41^###^	91.13±9.27^###^
Kidney cut + Fushen Decoction f	Initial	43.70±1.01	22.38±0.68	104.25±1.37	23.47±3.19
	2 weeks	94.04±3.57	22.56±5.68^#^	142.57±4.42	100.66±12.18^###, Δ^
	4 weeks	62.45±1.87^###^	23.48±1.12^#^	111.40±18.23^###^	178.82±12.34^###, Δ^
	6 weeks	62.45±1.87^###^	37.31±0.86^#^	93.28±3.56^###^	103.28±8.42^###, Δ^
Kidney cut + normal f	Initial	42.29±3.04	22.66±1.39	105.10±1.77	24.72±2.50
	2 weeks	98.63±0.32	24.57±1.40^#^	137.03±6.89^###^	83.47±8.82^###^
	4 weeks	57.04±2.81^###^	29.01±4.23^#^	63.47±1.93^###^	153.94±13.76^###^
	6 weeks	57.04±2.81^###^	43.92±0.51^#^	104.57±15.06^###^	98.73±7.51^###^

Compared with normal donor group,** P< 0.01,*** P< 0.001; compared with kidney cut + saline group, #P< 0.05, ### P< 0.001; compared with kidney cut + normal f group, Δ P > 0.05.

#### 4.2.3 Observation of serum endotoxins and metabolic toxin-like indicators of enteric origin

Compared to the normal donor group, the nephrectomy + saline group showed a statistically significant increase (P< 0.05) in IS, PCS, D-lactic acid, and endotoxin at weeks 2, 4, and 6, indicating successful modeling of renal failure. Compared with the nephrectomy + saline group, the nephrectomy + Fushen Granule group showed a significant decrease in PCS, D-lactic acid, and endotoxin at weeks 2 and 4 (P< 0.05), and a significant decrease in IS at week 6 (P< 0.05). Similarly, compared to the nephrectomy + saline group, the nephrectomy + normal f group showed significant decreases in PCS, D-lactic acid, and endotoxin at weeks 2, 4, and 6 (P< 0.05), and a significant decrease in IS at weeks 4 and 6 (P< 0.05). The improvement in D-lactate and endotoxin in the kidney cut + Fushen Granule group at weeks 2, 4, and 6 was not significantly different from that in the kidney cut + normal f group (P > 0.05) (**Table 8**).

**Table 8:** Indicators for D-lactate, endotoxin and enteric-derived metabolic toxin categories.

Group	Point in time	Metabolic toxins of enteric origin		Serum endotoxin	
		IS (μg/mL)	PCS (μg/mL)	D-Lactic acid (μg/mL)	Endotoxin (EU/ml)
Normal Donor	Initial	0.91±0.13	0.17±0.02	7.34±0.28	0.023±0.003
	2 weeks	0.93±0.06	0.15±0.1	7.52±0.36	0.028±0.003
	4 weeks	0.85±0.21	0.13±0.02	7.88±0.42	0.032±0.002
	6 weeks	0.86±0.23	0.15±0.01	7.52±0.28	0.029±0.004
Kidney cut + saline	Initial	0.83±0.12	0.14±0.01	7.58±0.19	0.030±0.004
	2 weeks	5.69±2.87**	1.73±0.22***	18.19±0.54^***^	0.387±0.032^***^
	4 weeks	5.24±0.73***	1.8±0.21***	15.28±1.25^***^	0.498±0.028^***^
	6 weeks	6.68±2.17***	2.12±0.62***	15.28±1.25^***^	0.518±0.028^***^
Kidney Cut + Fushen Decoction Donor	Initial	0.94±0.17	0.18±0.03	7.28±0.32	0.026±0.005
	2 weeks	4.98±1.25	1.28±0.31#	12.33±0.17^###^	0.224±0.021^###^
	4 weeks	4.83±1.00	0.37±0.06^###^	10.25±0.93^###^	0.243±0.017^###^
	6 weeks	3.64±1.10^#^	0.39±0.08^###^	10.25±0.93^###^	0.217±0.014^###^
Kidney cut + Fushen Decoction f	Initial	0.89±0.12	0.16±0.02	7.69±0.33	0.028±0.002
	2 weeks	4.90±1.31	1.03±0.19^##^	14.28±1.23^###, Δ^	0.258±0.028^###, Δ^
	4 weeks	4.22±0.92	0.93±0.15^###^	12.15±1.02^##, Δ^	0.305±0.020^###, ΔΔ^
	6 weeks	4.08±1.36^#^	0.86±0.21^###^	12.15±1.02^###, Δ^	0.293±0.021^###, Δ^
Kidney cut + normal f	Initial	0.86±0.09	0.15±0.01	7.21±0.21	0.027±0.003
	2 weeks	3.92±1.19	0.88±0.09^###^	13.57±0.67^###^	0.206±0.019^###^
	4 weeks	3.49±1.25^#^	0.75±0.11^###^	12.57±0.78^##^	0.251±0.019^###^
	6 weeks	2.78±0.64^##^	0.64±0.09^###^	12.57±0.78^###^	0.264±0.029^###^

Compared to the normal donor group, *** indicates P < 0.001; compared to the nephrectomy + saline group, # indicates P < 0.05, ## indicates P < 0.01, ### indicates P < 0.001; compared to the nephrectomy + normal f group, Δ indicates P > 0.05.

#### 4.2.4 Analysis of intestinal bacterial diversity

##### 4.2.4.1 Sequencing data quality and OTU analysis

**Figure 4:**
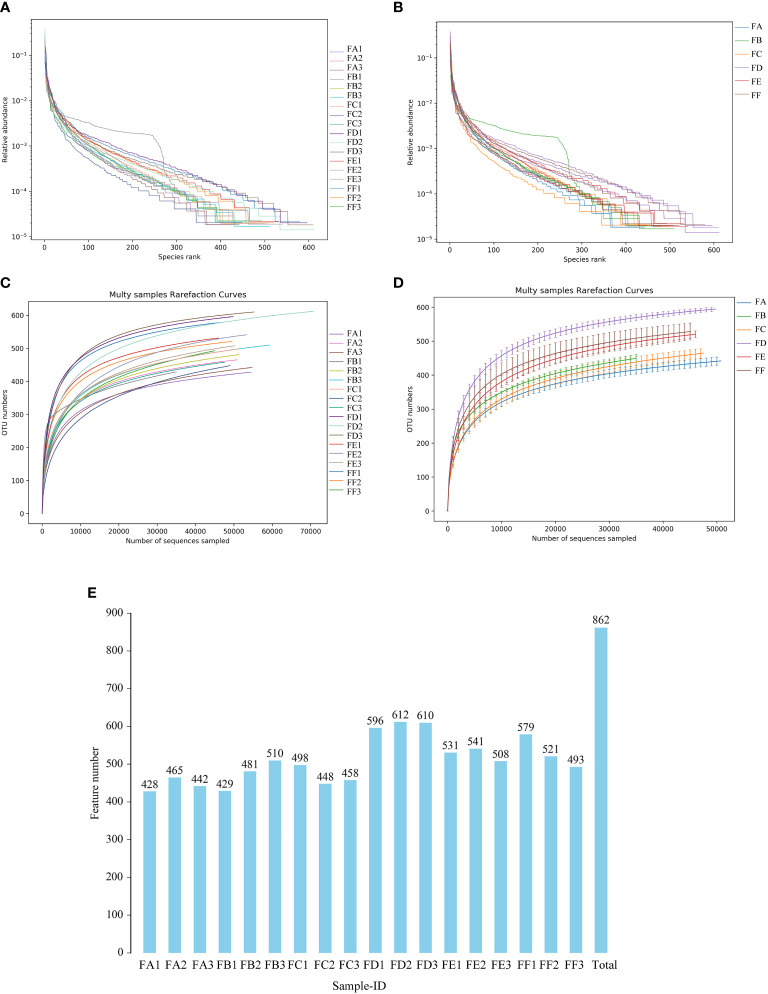
Single sample intestinal flora diversity analysis **(A, B)** Rank abundance curve The horizontal coordinate is the ordinal number sorted by the abundance of features, the vertical coordinate is the relative abundance of the corresponding features; **(C, D)** Sample dilution curve. The horizontal coordinate is the number of randomly selected sequencing strips, and the vertical coordinate is the number of features obtained based on the number of sequencing strips. Each curve represents a sample and is marked with a different color. **(E)** Plot of the number of features for each group of samples. The horizontal coordinate is the name of the sample, the vertical coordinate is the number of features, and the number above the bar is the number of OTUs for the corresponding sample.

In this study, the coverage of each sample library was assessed by calculating the Coverage Index, and values of the Coverage Index were obtained to be >0.99, indicating comprehensive coverage of each sample library and a high detection rate of the sample sequences (**Table 9**). Hierarchical abundance curves were used to simultaneously interpret the richness and evenness of the species contained in the samples. The graph shows that the distribution of microbial species in the sequenced samples, excluding sample B2, was reasonably even, it is possible that the B2 sample was contaminated during the sampling process, resulting in a significantly different species abundance and proportion from the pre-intervention samples (**Figures 4A, B**). Dilution curves were used to verify that the sequencing data reflected the actual biodiversity in the samples and indirectly the species richness in the samples. The results showed that the curve levelled off as the number of sequences increased, indicating that the depth of sequencing had largely covered all species in the samples (**Figures 4C, D**). In addition, the results of the number of OTUs of each sample obtained by clustering using usearch software showed that the number of rat intestinal flora characteristics in groups FD, FE and FF after Fecal microbiota transplantation(FMT) showed an overall increasing trend compared to groups FA, FB and FC before FMT. In terms of the number of rat intestinal flora characteristics after FMT, the kidney cut + normal group FMT (FD) > kidney cut + Fushen Granule FMT (FF) > kidney cut + saline group FMT (FE) (**Figure 4E**).

**Table 9:** Displays the analysis of intestinal flora diversity in each group of rats ( $\bar{\text{x}}$ ± s, n = 3).

Group	Chao	Ace	Shannon	Simpson	Coverage
FA	502.8182 ± 10.0702	495.2557 ± 9.7063	5.5293 ± 0.1972	0.9363 ± 0.0128	0.9987 ± 0
FB	564.9597 ± 10.163	569.3319 ± 19.4272	6.2448 ± 0.7023	0.951 ± 0.0204	0.9983 ± 0
FC	548.2892 ± 15.9462	535.5732 ± 13.0593	5.1845 ± 0.3452	0.9081 ± 0.0152	0.9983 ± 0.0003
FD	658.0036 ± 9.4833	645.3728 ± 9.0772	6.04 ± 0.5148	0.9244 ± 0.0399	0.9989 ± 0
FE	582.801 ± 7.0036	578.1836 ± 6.6149	5.6382 ± 0.4512	0.9257 ± 0.0281	0.9985 ± 0
FF	603.0205 ± 5.3112	583.5407 ± 15.2415	5.8779 ± 0.5683	0.9368 ± 0.0241	0.9984 ± 0.0005

FA before fecal microbiota transplantation(FMT) in the model group + Donor group; FB before FMT in the model group + control Donor group; FC before model group + saline intervention; FD after FMT in the model group + control Donor group; FE after model group + saline intervention; FF after FMT in the model group + Donor group.

##### 4.2.4.2 Alpha diversity analysis

Alpha diversity reflects the richness and diversity of species in a single sample and is measured by various indices such as Chao, Ace, Shannon, Simpson, Coverage, and peritoneal dialysis - whole_tree. The Chao and Ace indices measure species richness, i.e., the number of species, and larger values of the Shannon and Simpson indices are used to indicate the species diversity of a sample (Grice et al., 2009). Using QIIME2 software, the Alpha Diversity Index of the samples was assessed, and the values for each sample were tallied in **Table 9**. In terms of flora richness, the Ace and Chao indices increased in groups FD, FE, and FF compared to groups FA, FB, and FC, indicating an increase in flora richness in rats after fecal microbiota transplantation(FMT), with statistically significant differences. In terms of bacterial diversity, the Shannon and Simpson indices were used to estimate the diversity of the flora, and the higher the value, the higher the community diversity. It was found that the Shannon and Simpson indices in groups FD, FE, and FF were higher than those in groups FA, FB, and FC. This indicates that the diversity of the rat intestinal flora was restored after FMT. In conclusion, FMT helped to restore the richness and diversity of the rat intestinal flora. In the comparison between the groups, the normal group had the best results for FMT, followed by the FMT of Fushen Granule, and finally the saline sham operation group.

##### 4.2.4.3 Beta diversity analysis

**Figure 5:**
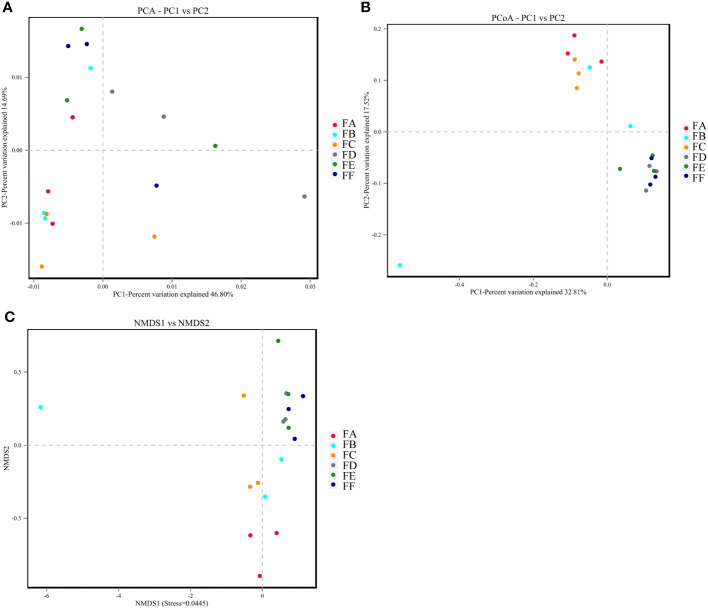
The beta diversity analysis. **(A)** PCA. The dots indicate each sample with different colors representing different groupings. The horizontal and vertical coordinates represent the two eigenvalues that cause the greatest variation between samples, with the percentage indicating the contribution of each principal component to the variation of the samples. **(B)** PCoA. Points represent each sample separately, with different colors representing different groupings. The horizontal and vertical coordinates represent the two characteristic values that cause the greatest variation between samples, and the degree of influence is represented as a percentage. The closer the samples are on the coordinate plot, the greater the similarity. **(C)** NMDS. The dots represent each sample separately, with different colors representing different groupings, and the distance between the dots indicates the degree of difference. A Stress value of less than 0.2 indicates that NMDS analysis has a certain degree of reliability, and the closer the samples are on the coordinate graph, the higher the similarity.

Beta diversity analysis was conducted in this study using QIIME software to compare the extent to which different samples were similar in terms of species diversity. Principal Component Analysis (PCA), Principal Coordinate Analysis (PCoA), and Non-metric Multidimensional Scaling Analysis (NMDS) were used to examine differences between samples. PCA uses variance decomposition to reflect differences between multiple data sets on a two-dimensional coordinate plot, with the axes representing the two eigenvalues that best reflect the variance. PCoA and PCA plots showed that groups FA, FB, and FC were closer together, indicating high consistency and less variability in the samples. On the other hand, samples FD, FE, and FF after 15 days were less similar in terms of species diversity compared to groups FA, FB, and FC, suggesting that the intervention caused a change in species variability. NMDS analysis had a reliable Stress value of 0.0445 (less than 0.2), indicating its accuracy. Overall, the results suggest that the transplantation of Fushen Granule and normal flora had a moderating effect on the changes in the composition of the intestinal flora in 5/6 nephrectomized rats (**Figures 5A-C**).

#### 4.2.5 Annotation and variability analysis of intestinal flora species

**Figure 6:**
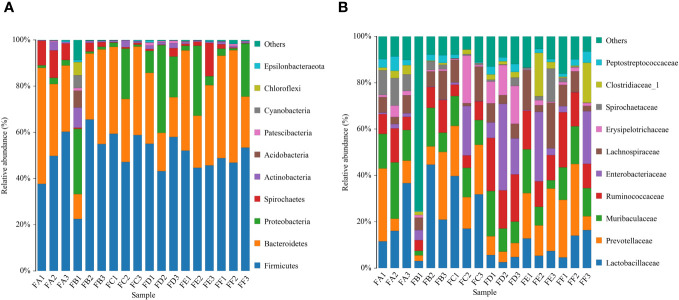
Histogram of the relative abundance of species composition of multiple samples for community composition analysis. **(A)** phylum level, **(B)** genus level. FA before FMT in the model group + Donor group; FB before FMT in the model group + control Donor group; FC before model group + saline intervention; FD after FMT in the model group + control Donor group; FE after model group + saline intervention; FF after FMT in the model group + Donor group.

The primary bacteria that dominate at the phylum level include firmicutes, bacteroidetes, proteobacteria, spirochaetes, actinobacteria, patescibacteria, tenericutes, cyanobacteria, and verrucomicrobia. The relative abundance of intestinal flora at the phylum taxonomic level was analyzed in **Figure 6**. Compared to rats before FMT, the model + saline group, the model + Fushen Granule Donor group, and the model + normal Donor group showed a decreasing trend for Firmicutes bacteria and an increasing trend for Bacteroidetes. Compared with the model + saline sham-operated group, the relative abundance of these two groups decreased less in the model + Fushen Granule Donor group and the model + normal Donor group, suggesting that the Fushen Granule and normal flora transplantation had a moderating effect on the relative abundance of colonic flora at the phylum level. At the genus level, the model group exhibited a decreasing trend of Lactobacillus after FMT. Compared with the Kidney-cut + saline group during the same period, the Kidney-cut + Fushen Granule Donor group and the Kidney-cut + normal Donor group had some degree of restorative effect on Lactobacillus, and the Fushen Granule FMT was superior to the other two groups.

#### 4.2.6 LefSe analysis

**Figure 7:**
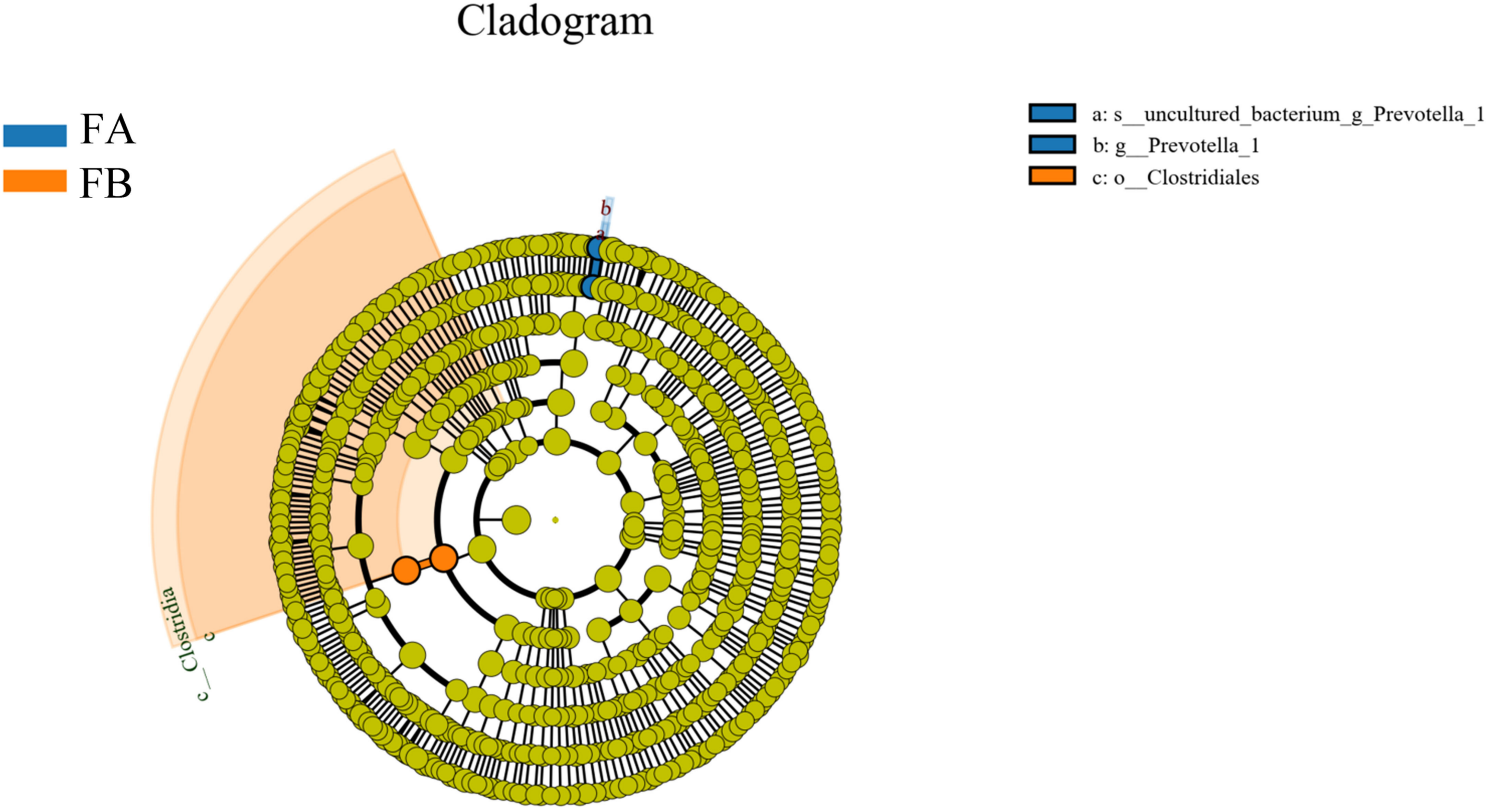
Evolutionary branching diagram for LEfSe analysis of the differences in the dominant groups. The circles radiating from the inside to the outside represent the taxonomic levels from phylum to species; each small circle at a different taxonomic level represents a taxon at that level, and the diameter of the small circles is proportional to the size of the relative abundance; the coloring principle is that species with no significant differences are colored uniformly in yellow, and other differing species are colored according to the subgroup in which the species is most abundant. Different colors indicate different subgroups, and different colored nodes indicate the microbiota that play an important role in the subgroup represented by that color.

In order to screen for colonies with significant differences between groups, this study employed a novel metagenomic analysis method called LefSe (Linear Discriminant Analysis Size Effect). This method is a statistical approach used in the fields of genetics and microbiology to identify high-dimensional biomarkers and reveal genomic features. LefSe analysis combines linear discriminant analysis with non-parametric tests to identify multiple biomarkers and enable comparisons between multiple groups. In this study, LDA values greater than 3.5 were used as screening criteria to examine the abundance of the flora. In an evolutionary branching tree diagram, different circles represent various taxonomic levels from inner to outer, including domain, kingdom, phylum, class, order, family, genus, and species. Different nodes in the diagram represent dominant genera, while the connections between nodes indicate their correlation. The thickness of the lines reflects the strength of the correlation; thicker lines represent stronger correlations. Additionally, the size of the nodes represents the number of associations with other microorganisms; the more associations, the larger the node. The results showed that Model Group FA had significant differences in a) uncultured_bacterium_g_Prevotella_1 and b) g_prevotella_1. Group FE exhibited significant differences in c) o_Clostridiales after FMT (**Figure 7**).

## 5 Discussion

As a progressive, irreversible disease with a complex pathogenesis, limited therapeutic modalities, and issues of tolerance and dependence, the development of effective drugs to treat or alleviate CRF has become an urgent task (Eirin and Lerman, 2014). The gut’s microbial composition is a key player in renal disease and is closely related to the development of CRF (Meijers et al., 2019; Zhu et al., 2021; Krukowski et al., 2023). Based on our findings, the use of Fushen Granule can regulate the intestinal microbiota and control the progression of CRF. And we demonstrated that FMT in rats in the Fushen Granule group reversed the severe taxonomic and functional imbalance caused by CRF to a certain extent. This represents the first published study to date utilizing the combined treatment of Fushen Granules and FMT for CRF. The Fushen Granule and its compound “Fushen Granules” have been used clinically in the treatment of various kidney diseases such as peritoneal dialysis and interstitial fibrosis (Chen, 2021; Ding, 2022). Modern pharmacological studies have also shown that Astragalus, the primary component of Fushen Granule, has the ability to reduce urinary protein (Zhang et al., 2014) and protect kidney function (Zhou et al., 2017). The combination of Astragalus and Salvia miltiorrhiza can slow down kidney fibrosis (Han et al., 2021) and suggests a high level of drug safety. *In vitro* and clinical trials have demonstrated that Fushen Granule can, to a certain extent, correct intestinal dysfunction in peritoneal dialysis patients and delay the process of peritoneal fibrosis. Meanwhile, there is increasing evidence suggesting that traditional Chinese medicine plays a beneficial role in the treatment of chronic kidney disease by modulating dysbiosis of the intestinal microbiota (Gao et al., 2021; Ming et al., 2021). However, the effect of Fushen Granule on CRF and the specific potential mechanism remain to be elucidated. Therefore, in this experiment, the effect of Fushen Granule on CRF rats was investigated using a 5/6 nephrectomized CRF rat model. The experimental results showed that the basic conditions of CRF rats were alleviated after different doses of Fushen Granule, and the levels of the intestinal toxins indoxyl sulfate (IS) and p-cresyl sulfate (PCS), as well as D-lactic acid and endotoxin, which are indicators of intestinal barrier damage, were reduced in a dose-related manner. At the same time, the number of operational taxonomic units (OTUs), the ratio of Firmicutes to Bacteroidetes, and the number of lactic acid bacteria increased after Fushen Granule treatment, which restored to some extent the changes in the abundance and structure of the intestinal flora of the rats caused by slow renal failure.

CRF is accompanied by a progressive decrease in glomerular filtration rate and the continued accumulation of metabolic waste products that can accelerate the progression of CRF and affect its prognosis. The enteric-derived toxins indoxyl sulfate (IS) and p-cresol sulfate(PCS) are known as protein-bound toxins due to their protein-binding properties and are currently not filtered out by dialysis. In normal renal function, these two substances are secreted into the renal tubules and excreted from the body. In CRF, the intestinal tract undergoes a series of changes, including a lack of gastrointestinal motility and reduced digestive capacity of the small intestine, which increases the amount and retention time of dietary protein in the colon, which further contributes to the increase in the number of colonic proteolytic bacteria, resulting in increased production of the IS and PCS precursors indole and p-cresol, and ultimately an increase in IS and PCS generation. At the same time, renal filtration function is reduced, resulting in the accumulation of metabolic toxins such as IS and PCS in the body. The long-term retention of IS and PCS in the body can further aggravate glomerulosclerosis and renal fibrosis, leading to a vicious circle (Ito et al., 2010; Lekawanvijit et al., 2010; Niwa, 2010; Liabeuf et al., 2011; Shimizu et al., 2011; Lin et al., 2012; Liu et al., 2012). Therefore, reducing the level of IS and PCS is one of the most important problems to be solved. There is a lack of effective means of IS and PCS clearance, and most of the research on reducing IS and PCS has been conducted in the intestinal tract from the perspective of inhibiting their production, such as with AST120 (Shimoishi et al., 2007; Kikuchi et al., 2010; Asai et al., 2019). Since enteric-derived toxin precursors are primarily produced by the enzymatic processes of the intestinal flora, it is possible to reduce their production by adjusting the structure of the intestinal flora. In the present study, Fushen Granule demonstrated a notable down-regulating effect on the levels of intestine-derived metabolic toxins IS and PCS in CRF rats. It is hypothesized that the mechanism may be related to improving the intestinal tract status and promoting the excretion of toxin precursors.

Serum endotoxin is a metabolite or component of intestinal bacteria that can translocate into the circulation when the barrier is not functioning properly (Violi et al., 2023), it is used as an indicator of intestinal barrier function (Vancamelbeke and Vermeire, 2017). Fushen Granule can improve renal function indicators by repairing intestinal barrier function, reducing metabolic toxins in the serum, and enhancing renal function. D-lactate is a bacterial metabolite produced by the intestinal flora, and its level is low and relatively stable under normal conditions. Elevated levels may reveal the extent of damage to the intestinal mucosa (Guo et al., 2019). Endotoxin, a major component of the outer membrane of Gram-negative bacteria, can translocate into the body’s circulation when intestinal barrier function is compromised, transforming host immune defenses into a pro-inflammatory state (Violi et al., 2023). The endotoxin itself can further contribute to the deterioration of the mucosal barrier function (Tang et al., 2019). In this study, serum endotoxin and D-lactate levels were measured in CRF rats to assess intestinal barrier function. In the sham-operated group, serum endotoxin levels were stable, whereas in the model group, serum endotoxin levels increased with the progression of CRF. The model rats also showed a significant increase in serum D-lactate, both of which are indicative of impaired intestinal barrier function. Fushen Granule reduced the variation of endotoxin and down-regulated the serum D-lactate level, which had a protective effect on the intestinal barrier function and thus delayed the progression of CRF.

Changes in the intestinal flora play a key role in accelerating the progression of CRF, and this study found that Fushen Granule was able to modify the structure of intestinal flora in rats to slow down the progression of CRF. Previous research has reported that in patients with ESRD, the number of Firmicutes, Actinobacteria, Proteobacteria, and Lactobacillus decreased( Vaziri et al., 2013; Simões-Silva et al., 2020), while the number of Bacteroides increased (Crespo-Salgado et al., 2016). In patients with CKD, there was a lower abundance of Lactobacillus and an increased proportion of Enterobacteriaceae (Mahmoodpoor et al., 2017; Lau et al., 2018). It is evident that there is an imbalance between the intestinal flora and the host in the disease state, which may lead to the accumulation of uremic toxins while limiting the beneficial functions and products conferred by the normal flora, thereby accelerating the progression of renal disease (Vaziri et al., 2013). In contrast, the homeostatic gut microbiota serves the host through a wide range of physiological activities, including the prevention of pathogens, maintenance of the function and integrity of the intestinal epithelium, and regulation of the host immune system (Bian et al., 2022). 16S rRNA or DNA sequencing is the common method to evaluate microbial diversity and identify differential microbes in patients compared with healthy control subjects (Cao et al., 2022). The present study, combined with 16S rRNA gene sequencing experiments, aimed to investigate the mechanism of action of Fushen Granule in improving CRF from the perspective of structural changes in the intestinal flora. The results showed that the rats in the model group exhibited a decrease in the number of OTUs in the intestinal flora. At the phylum level, the relative abundance of bacteria in the Firmicutes phylum decreased in the model group after 5/6 nephrectomy compared to the sham-operated group during the same period, while the content of the Bacteroidetes phylum increased, and the ratio of the Firmicutes phylum to the Bacteroidetes phylum showed a decreasing trend. After Fushen Granule intervention, the low-dose and high-dose groups of Chinese medicine reduced this alteration to different degrees, thus restoring the normal ratio of Firmicutes and Bacteroidetes to a certain extent and repairing the alteration of the composition ratio of intestinal flora caused by slow renal failure. At the genus level, the relative abundance comparison showed that Lactobacillus was the predominant genus. This genus is a probiotic strain that can slow down the progression of kidney disease by improving the intestinal environment (Yoshifuji et al., 2016). The relative abundance of Lactobacillus in the model group showed a decreasing trend compared to the sham-operated group during the same period. In contrast, the relative abundance of Lactobacillus in the low-dose and high-dose groups of Chinese medicine showed an increasing trend after treatment. This indicates that Fushen Granule can, to a certain extent, reverse the effect of 5/6 nephrectomy on the intestinal flora of rats, correct the disorder of flora, and make it converge to the normal group, and has the effect of improving the intestinal flora of CRF rats. In conclusion, the restoration of the altered intestinal flora in the CRF state by Fushen Granule may be another mechanism of its action in delaying renal failure.

To further investigate whether Fushen Granule exerts its therapeutic effect through the regulation of intestinal flora, a FMT experiment was conducted to verify the effect of Fushen Granule. Multiple studies indicate that the transplantation of a healthy gut microbiota plays a therapeutic role in various renal diseases. A clinical case study report suggests that FMT reduces the accumulation of PBUTs in the host by modulating the intestinal microbiota’s amino acid metabolism, consequently mitigating the progression of CKD (Liu et al., 2022). In investigations on IgAN, it has been reported that FMT-induced microbial transfer regulates the IgAN phenotype, thereby paving the way for new treatment modalities for IgAN patients (Lauriero et al., 2021). Furthermore, in various published studies, FMT has consistently been a valuable tool in verifying the correlation between gut microbiota dysbiosis and the progression of CRF, indicating its value in restoring the intestinal microbiota in CRF. However, there is currently limited understanding of the application of FMT in the treatment of CRF. In this study, we used a rat model to comprehensively evaluate the effects of Fushen Granule on the improvement of serum intestinal endotoxins, intestinal flora structure and diversity, and renal function in CRF with the aid of FMT technology. The results of the study showed that both the Fushen Granule Donor Group and the FMT Group could effectively improve the general status of CRF rats, enhance renal function, reduce serum levels of toxins, and improve intestinal status to promote excretion of toxin precursors, reduce D-lactate, narrow the rise of endotoxin, and protect intestinal barrier function after treatment. Alpha and beta diversity were assessed in the feces collected from each group of rats at the end of fecal microbiota transplantation(FMT). Alpha diversity was assessed mainly from the Ace, Chao, Shannon, and Simpson indices. The results showed that all the above indices exhibited different degrees of increase in the model group after FMT, suggesting that the diversity of rat intestinal flora was restored and the abundance tended to increase. The Ace, Chao, Shannon, and Simpson indices of the model + control Donor group and the model + Fushen Granule Donor group were very close to each other compared to the model + saline sham group. β diversity was demonstrated by PCA, PCoA, and NMDS, and the results showed that there was little overlap between the three groups, suggesting significant differences in the structure of the flora between the groups. Further analysis of the composition and species abundance of the intestinal flora of each group revealed that Fushen Granule and normal flora transplantation had a moderating effect on the structure and relative abundance of the intestinal flora of CRF rats.

## 6 Conclusion

Based on the 16SrRNA amplification and sequencing technology, this study investigated the mechanism of action of Fushen Granule in the prevention and treatment of CRF in rats with 5/6 nephrectomy as a classical renal failure animal model. The results showed that Fushen Granule was effective in the treatment of CRF rats, potentially delaying the process of CRF by repairing intestinal barrier function, reducing metabolic toxins in serum, improving renal function indicators, and adjusting the structure of intestinal flora. In addition, through FMT experiments, we found that the transplantation of feces from the Fushen Granule Group was able to replicate the intestinal flora structure of the original Fushen Granule Group to a certain extent in the subject rats, and improve the renal function and intestinal status of the CRF rats. This further demonstrates that Fushen Granule can delay the progression of CRF by regulating the “renal-intestinal axis” and improving the intestinal flora structure. This study demonstrates that the Fushen Granules are advantageous in enhancing the structure of the intestinal microbiota and play a constructive role in the prevention and treatment of CRF, thereby offering a scientific basis for its clinical application. The results also suggest that reshaping the homeostasis of the intestinal flora may be an important therapeutic target to slow down the progression of CRF.

## Data availability statement

The original contributions presented in the study are included in the article/supplementary material. Further inquiries can be directed to the corresponding author.

## Ethics statement

The animal study was approved by Institute of Radiology, Chinese Academy of Medical Sciences. The study was conducted in accordance with the local legislation and institutional requirements.

## Author contributions

LW: Methodology, Visualization, Writing – original draft. AX: Software, Writing – original draft. JW: Software, Writing – review & editing. GF: Writing – review & editing. RL: Formal analysis, Methodology, Writing – review & editing. LJW: Data curation, Writing – review & editing. MP: Conceptualization, Funding acquisition, Writing – review & editing.
